# Reliability and reproducibility of disc-foveal angle measurements by non-mydriatic fundus photography

**DOI:** 10.1371/journal.pone.0191007

**Published:** 2018-01-25

**Authors:** Caroline Le Jeune, Fayçal Chebli, Lorette Leon, Emmanuelle Anthoine, Michel Weber, Alain Péchereau, Pierre Lebranchu

**Affiliations:** 1 Department of Ophthalmology, University Hospital of Nantes, Nantes, France; 2 Department of Ophthalmology, Docteur Nekkache Hospital, Algiers, Algeria; 3 Clinic of Ophthalmology (CNO), Neuchâtel, Switzerland; 4 Department of Public Health, University Hospital of Nantes, Nantes, France; 5 UMR 6004 CNRS, Image Perception and Interaction Team, Laboratoire des Sciences du Numérique de Nantes (LS2N), Nantes, France; Washington University in Saint Louis, UNITED STATES

## Abstract

**Purpose:**

Abnormal torsion could be associated with cyclovertical strabismus, but torsion measurements are not reliable in children. To assess an objective fundus torsion evaluation in a paediatric population, we used Non-Mydriatic Fundus photography (NMFP) in healthy and cyclovertical strabismus patients to evaluate the disc-foveal angle over time and observers.

**Methods:**

We used a retrospective set of NMFP including 24 A or V-pattern strabismus and 27 age-matched normal children (mean age 6.4 and 6.7 years respectively), taken during 2 distinct follow-up consultations (separated by 251 and 479 days respectively). Each disc-foveal angle measurement (from which the ocular torsion can be assessed) was performed by 5 different observers, using graphical software and based on reproducible fundus anatomical marks. Statistical analysis was performed with a multivariate ANOVA using group, time and observers as factors, in addition to intraclass coefficient correlation (ICC) to assess measurement reproducibility.

**Results:**

A significant difference of disc-foveal angle measures was observed between groups (p<0,001): 18.73° (SD = 6.42), -3,25° (SD = 5.51) and 6,89° (SD = 4,41) respectively for V-pattern, A- pattern and normal subjects. Neither observers (F = 0,2028 p = 0,9369) nor time between 1^st^ and 2^nd^ NMFP (F = 0,6312 p = 0,4271) seem to influence the measure of disc-foveal angle. The evaluation of disc-foveal angle was very reproducible between observers (ICC>0,97).

**Conclusion:**

Abnormal amount of objective torsion could be associated with alphabet-pattern strabismus. Disc-foveal angle evaluation by NMFP in a children population appears as a non-invasive, reliable and reproducible method.

## Introduction

Measuring cyclotorsion in everyday strabismological practice is a useful tool, used since 1840. Abnormal cyclotorsion can be observed after brutal imbalance between vestibular impulses as in skew deviation or acute unilateral palsy of an oblique muscle [1,2]. Abnormal disc-foveal angle is also implicated in A and V-pattern strabismus both for diagnosis and to dictate different surgical approaches [3–5].

In order to assess cyclotorsion, several subjective methods have been described. An indirect way consists of analysing the excursion of the blind spot on the Goldman perimetry. Torsion can also be assessed using Krats torches during a coordimetric Lancaster red-green test, with Harms’ tangent scale, with Bagolini’s striated glass or the double Maddox rod [6,7]. All of these tests require a normal binocularity and a perfect cooperation of the subject. They are not reliable in case of abnormal retinal correspondence or severe amblyopia. Moreover there is a subjective sensory adaptation to fundus torsion with time, particularly if the cyclotorsion appears early during childhood [8].

Cyclotorsional analysis in case of abnormal sensoriality, particularly in very young children, requires the development of more objective methods. Using anatomical ocular landmarks is a valuable objective method. If anterior segment photographs enable good dynamic cyclotorsion analysis, its anatomical diversity does not allow inter-individual comparison. On the other side anatomical landmarks of the retina, fovea and optic disc, seem relatively constant, and disc-foveal angle can be measured with fundus photography [9].

Non-Mydriatic Fundus photography (NMFP) has the advantage of being a non-invasive test, easily and quickly performed, especially in young patients. Normative values between the fovea and the center of the head of the optic nerve are ranging from 5.6° (+/-3.3) to 7.3° (+/-2.6), and do not seem to vary with age [9–11]. Objective cyclotorsion in pathological condition has not been extensively studied. A slight increase of excyclotorsion has been observed in case of unilateral 4^th^ nerve palsy, both in the paretic (10.7°) and in the non-paretic eye (8.8°) [12]. Abnormal excyclotorsion (13.4° +/- 6) or incyclotorsion (14° +/- 7) has been observed in case of inferior or superior oblique overaction [13]. Abnormal torsion has been associated with alphabet-pattern strabismus, but only in a qualitative way of measures [5]. No quantitative data is actually available in such cases.

NMFP appears as a reliable method to assess objective torsion, but there is few data about its reproducibility over time, and observers. We propose to use this method to assess disc-foveal angle in a paediatric population. We compared children known to present abnormal cyclodeviation (pattern strabismus with apparent oblique overaction) with an age-matched control group. Measurements were extracted from different NMFP obtained in two successive consultations and analysed by 5 different observers to check the repeatability and the variability of the method. We found abnormal statistical values of torsion in this particular group of strabismus, giving objective reference for follow-up and treatment. Cyclotorsion does not change statistically overtime and over observers.

## Methods

### Patients

We used a retrospective set of data, including patients between 2003 and 2011 in the ophthalmology department of Nantes University Hospital. The protocol was approved by the local ethic committee (Groupe d’Ethique Nantais dans le Domaine de la Santé: GNEDS, Nantes University Hospital, Ref: RC11_0064) and followed the tenets of the declaration of Helsinki.

The pathological group was identified by a retrospective review of all children between 3 and 11 years of age operated for pattern strabismus (n = 81). Only those with the inclusion criteria were included (n = 24): pattern strabismus, apparent oblique over-action, no previous surgery, and at least 2 NMFP available in the chart before surgery. All of them had reliable anatomical fundus landmarks, without ectopic fovea. The exclusion criteria were as a result of other associated ophthalmologic pathologies.

The control group (n = 27) was built with age-matched healthy children consulting for systematic ophthalmopeadiatric examination between January and August 2011. Inclusion criteria were normal visual acuity (48/60 or more), no previous history of ocular disease (including no strabismus), normal stereoscopic screening test (Lang I), normal fundus exam and at least 2 NMFP available in the chart.

In all cases, the two fundus photography had to be done at two different consultations.

All patients underwent a full oculomotor examination by an experienced ophthalmologist. Relevant clinical data for this study included angular deviation in primary position, in up and down gazes. Angular deviation was measured at distance fixation, with optical correction prescribed after cycloplegia for at least one month in case of pattern strabismus, with alternate cover test and prism (or Krimsky method if impossible). V-pattern was defined as a positive difference of 15 prism diopters (Δ) or more between down gaze and up gaze. A-pattern was as a negative difference of 10 Δ or more between down gaze and up gaze. Apparent oblique overaction and dissociated vertical deviation (DVD) were noticed if present.

### Non-Mydriatic Fundus photography

We used a previously described methodology and protocol to perform and analyse Non-Mydriatic Fundus photography [11]. As for every patient, subjects were installed in front of the non-mydriatic retinograph (Canon CRDGi) in a dim light room. They were asked to stay in the same position with the head maintained straight by a chin and forehead rest. The pictures were recorded as « JPEG » file and transferred to a personal computer.

The pictures were then analysed by the Adobe Photoshop Elements software. Three main marks were chosen on the pictures to calculate the disc-foveal angle δ: the center of the fovea (a), the superior edge (b) and the inferior edge (c) of the optic disc. Using previously described formula, 3 angles β, γ and δ were calculated [11].

Using these 3 references, we calculated the torsional angle δ between the middle of the optic nerve, the fovea and the horizontal line crossing it:

δ=((arctan((Yb‑Ya)/(Xb‑Xa)*180/π)+(arctan((Yc‑Ya)/(Xc‑Xa)*180/π))/2

**Fig 1:**
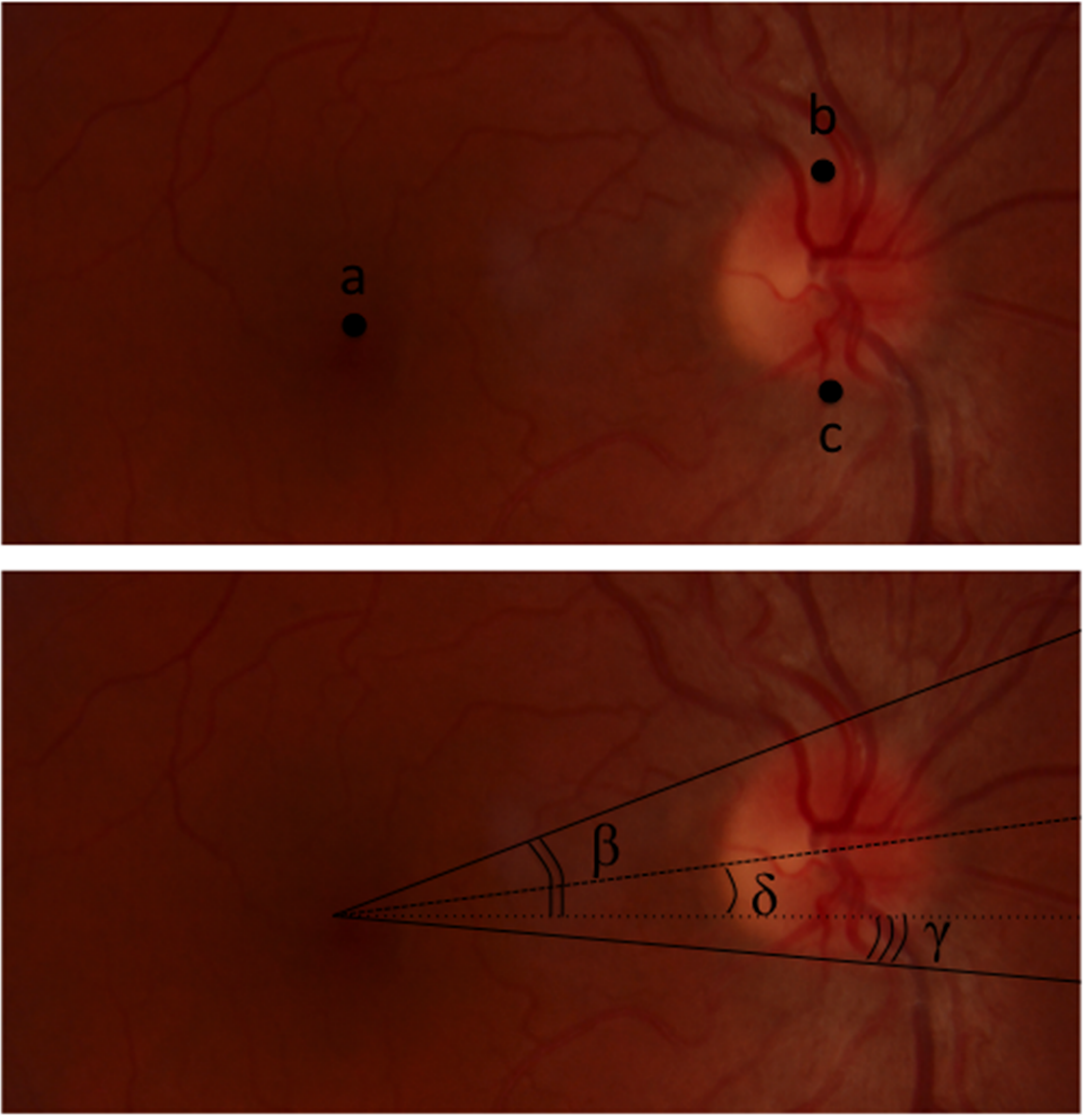
**Summary of the 3 main marks (a, b and c) chosen to calculate the disc-foveal angle on the NMFP and of the 3 angles measured by trignometric formulas.** δ represents the disc-foveal angle angle δ = (β+γ)/2.

By convention, when the middle of the optic nerve was above the horizontal line crossing the fovea, the angle value was positive (excyclotorsion); when it was below, the value was negative (incyclotorsion). (Fig 1)

### Data analysis

For each NMFP the measurement of the disc-foveal angle was practiced by five different observers: two senior ophthalmologists, one fellow ophthalmologist and two orthoptists students.

Data analysis was performed using JMP9 software. Qualitative data (gender, groups of subjects) was compared using a Chi square test. A multivariate ANOVA (completed by a post-hoc Tukey test if necessary) was performed to search for any statistical difference in disc-foveal angle measures according to groups, eye laterality, follow up or observers.

Intraclass coefficient correlation (ICC) between 5 observers was calculated for one laterality eye and one NMFP. The calculation was reproduced for the two NMFP on each laterality.

### Surgery effect

Post operative NMFP was analysed by one observer (observer 5) for A and V pattern patients after surgery. Differences between pre and post operative cyclotorsion and between post operative and control group cyclotorsion were determined by a non parametric Wilcoxon test.

## Results

24 children with strabismus (19 with V-pattern strabismus and 5 with A-pattern strabismus) and 27 healthy children were enrolled in the study. Mean age was 6 (from 3,8 to 11,4), 6.4 (from 4,5 to 11,7) and 6.7 (from 3 to 11,4) respectively for V-pattern, A-pattern and control group. There was no statistical difference in the children’s age (*F*(1,49) = 0.85; p = 0.36) or in the children’s sex ratio (Chi2 = 2.32, ddl = 1; p = 0.13).

The mean period between the 2 distinct NMFP was 251 days (from 92 to 505 days) in the strabismus group and 479 days (from 8 to 2188 days) in the control group. A significant difference in the time between the 2 NMFP was observed, with a longer time for the healthy children (*F* (1, 49) = 4.81; p = 0.03).

The mean disc-foveal angle was significantly different between the strabismus and healthy group (F = 903,7441 p<0,001), with 18.73° (SD = 6.42), -3,25° (SD = 5.51) and 6,89° (SD = 4,41) respectively in V-pattern, A- pattern and normal groups. The mean disc-foveal angle was also significantly different between left and right eye (F = 14,6097, p<0,0001), respectively 11,3676° and 9,2511°.

Comparison of disc-foveal angle measurements on each NMFP, between the 5 different observers does not reveal significant difference (F = 0,2028 p = 0,9369).

**Fig 2:**
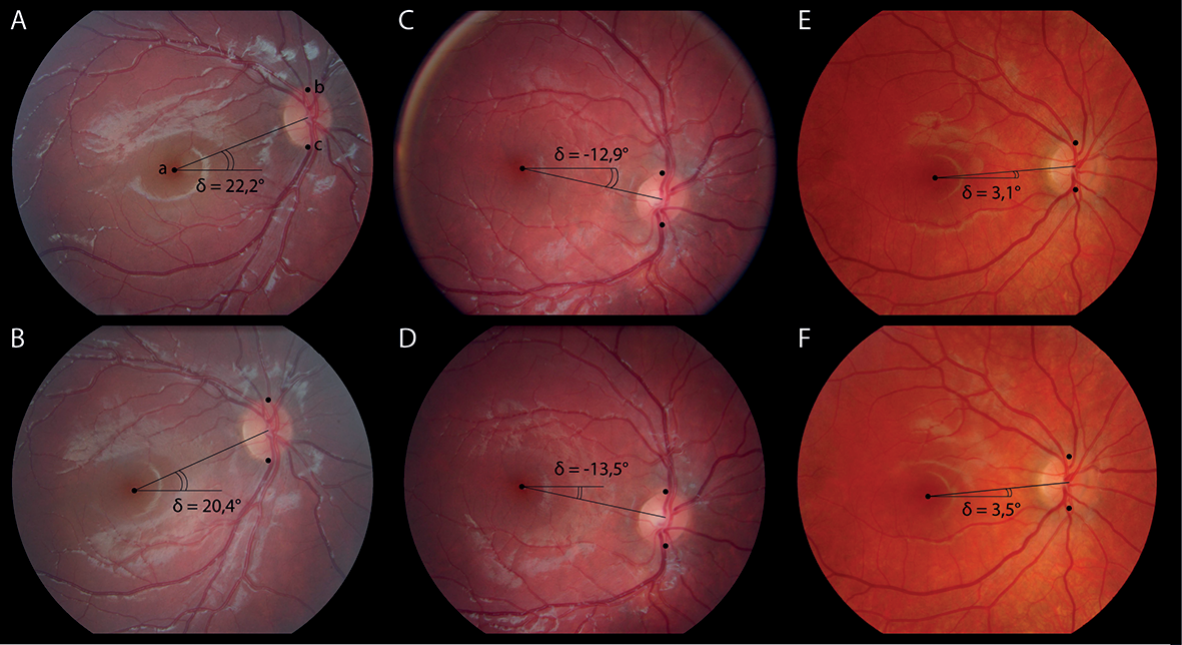
Examples of disc-foveal angle stability between NMFP 1 and NMFP 2 in a V-Pattern strabismus (A and B), a healthy child (C and D) and an A-Pattern strabismus (E and F).

**Fig 3:**
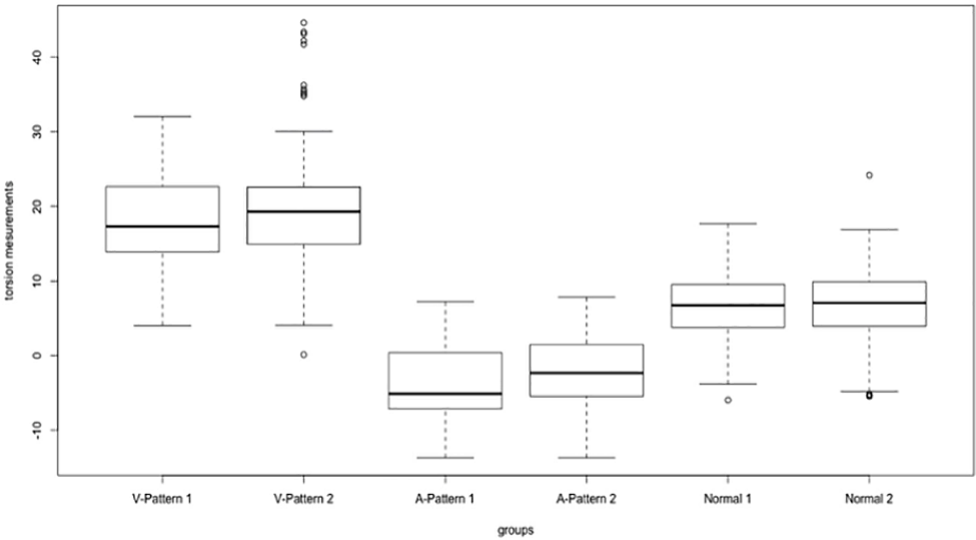
Boxplots representing variability of disc-foveal angle measurements (in degrees) over times: NMFP 1 and NMFP 2 and subgroup: V-Pattern, A-Pattern and healthy children.

No significant difference was observed between the first and the second NMFP (F = 0,6312 p = 0,4271). (Figs 2 and 3)

The ICC calculation found an excellent concordance (> 0,80) between the 5 observers regardless of eye laterality or numbers of NMFP; for 1^st^ NMFP and right eye ICC = 0,97, for 1^st^ NMFP and left eye ICC = 0,97, for 2^nd^ NMFP and right eye ICC = 0,96 and for 2^Nd^ NMFP and left eye ICC = 0,97.

Surgery significantly reduces the abnormal cyclotorsion in A-pattern (mean = 3.4°, SD = 5.2, p = 0.002) and V-pattern groups (mean = 9°, SD = 4.6, p<0.001). However post operative cyclotorsion significantly remains different from group control torsion (p = 0.02 for V-pattern group, p = 0.04 for A-pattern group).

## Discussion

**Fig 4:**
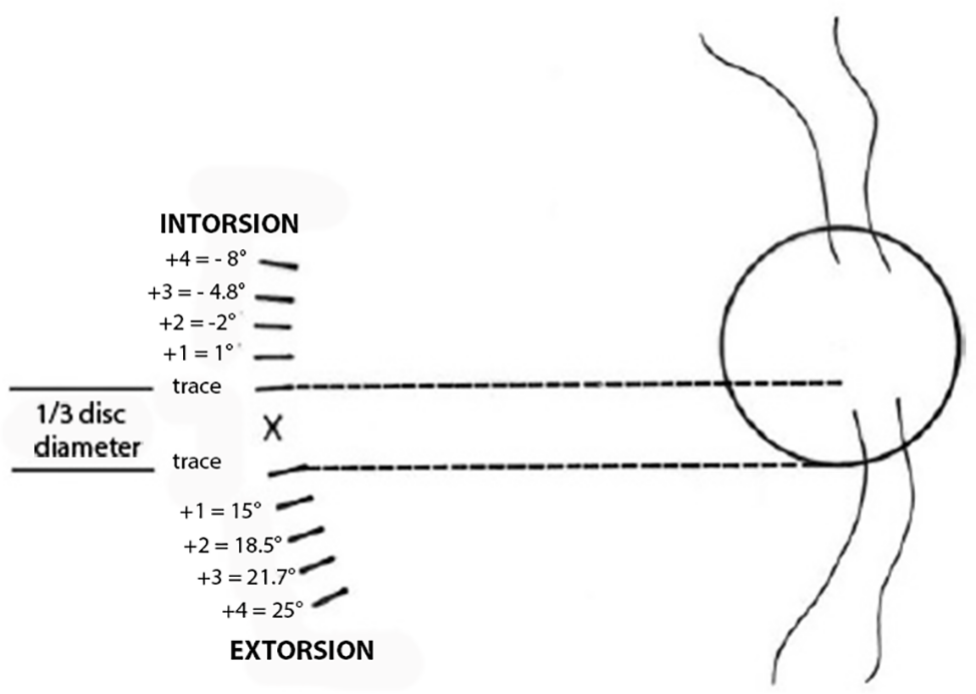
Modification of the figure from Guyton including the disc-foveal angle values.

Most of clinicians use qualitative methods to quantify abnormal cyclotorsion. For example, the disc-foveal angle measurement to the -4 to +4 scale could be commonly used in estimation of fundus torsion from the indirect ophthalmoscopic view [3]. Using the original picture of this scale and our method of torsion measurement allowed us to give a quantitative evaluation of this scale: respectively -8°, -4,8°, -2° and 1° for qualitative -4 to -1 incyclotorsion, and +15°, +18.5°, +21,7° and +25° for qualitative +1 to +4 excyclotorsion. (Fig 4)

Torsion measurement has been studied extensively over the past 40 years, based on subjective perception of patients or objective ocular landmarks with methods as new as the scanning laser ophthalmoscopy (SLO) fundus cyclometry or using vascular cues [6,14–16]. However, no study has assessed the reliability and reproducibility of this measure, especially among a paediatric population. The use of SLO is limited in children both because of the difficulty of a long fixation time and the limited access to the equipment. Using the vascular cues to assess objective torsion could be an effective method in case of obscured fovea or abnormal optic disc anatomy, but measures seem to be less precise [17]. Moreover it could sometimes be difficult to judge the center of the fovea in fundus photographs. This problem could be solved by the use of a pointer at the time of the camera acquisition. The pointer should be also visible on the fundus photography. Moving this pointer along the horizontal axis and asking the subject to follow it allowed the examiner to see exactly the level of the fovea relative to the position of the optic disc.

Our results easily gave measurements of disc-foveal angle in the paediatric population. It confirms that the mean objective torsion in our A or V-pattern strabismus group was statistically different from the one measured in healthy children. Objective torsion in the 4^th^ nerve palsy ranged between 11° and 14°, less than the 18.7° objective measure in the V-pattern group, probably reflecting different causes of cyclodeviation [12,18]. The incyclotorsion in the A pattern group (-3,2°) was smaller than the -14° objective torsion observed by Kushner in superior oblique muscle overaction [13]. The small number of pathological patients included in our A pattern-group could have biased our results, and repeated measures in a wider group are needed.

Our normal group had a physiological disc-foveal angle of 6.89°± 4.41°, in agreement with previous reports: 7.25°±2.6°, 6.3°± 3.4° and 6.1°±4.3° respectively [9,11,19]. Interestingly only one of these studies reports a normal value in a group of 50 children, with similar results [11]. Objective torsion seems not to vary with age.

Our results confirmed the reliability and reproducibility of this non-invasive objective method to measure disc-foveal angle. First the disc-foveal angle does not change with the duration of the follow up in each group of subjects. Secondly we point the reproducibility of the measurements without difference between observers, and an excellent ICC. Although the set of NMFP data has been done on retrospective review of charts, it should not influence the inter-observers analysis. However, a prospective study is needed to confirm the significant results between groups.

Stability of inter-observers measurements over time encourages the clinical use of measuring objective cyclotorsion using NMFP. Alphabet-pattern strabismus surgery is usually based on prismatic measures, but literature reports huge possibilities for procedures, including horizontal or vertical displacement of recti muscle insertion, oblique muscle recession or resection or anteroposition [20–23]. Objective measure of torsion could be a new informative data, helping to isolate sub-group with abnormal fundus torsion. It has also been proposed as an objective measure to check surgical efficiency [24]. Using NMFP to measure changes in disc-foveal angle according to head position could also help to answer this challenge in refractive surgery [25,26].

## Conclusion

NMFP has an important role in our daily practice. It helps the clinician for diagnosis, follow-up and management of some patients with strabismus. It easily gives an objective measure of disc-foveal angle. This method based on standardized parameters is reliable and reproducible in a population of children, and is not influenced by the observers. It allows new quantitative data, especially in case of abnormal torsion of the eyeball as in cyclovertical strabismus. Adding this objective data to prismatic measurements could give a new objective target to assess therapeutic strategies for strabismus.

## Supporting information

S1 Table All Torsion measurements of the 5 observers, for each NMFP (1 and 2) for right and left eyes, V-Pattern, A-Pattern strabismus and healthy children. (XLSX)

S2 Table All pre and post operative torsion meausurements of observater 5. (XLSX)
